# 

**Published:** 1855-09

**Authors:** 


*To Correspondents.*—Our present number is so filled with original matter, as to exclude most of the eclectic department. The value of the original articles is such, however, as to leave us no regrets on this score. The paper on opacities of the cornea, by Dr. Glück, is one of unusual merit. It will be concluded in our next. That upon medical education, by Dr. Chandler, is equally welcome.

We allude to these, especially, as coming from new contributors of established reputation as writers. It will be our aim to increase the number of our able collaborators, in the conviction that thus only can we make a good journal.

For our next number we have, besides the continuation of Dr. Glück's article, a case from Prof. White, of spina bifida of the anterior fontanelle, illustrated by a beautiful lithograph from Compton; a case of cyanosis, from Dr. E. Delany, of Fond-du-lac, Wisconsin; and an analysis of cases of onychia, from Mr. Austin Flint, Jr., of this city.

We congratulate ourselves upon the high character of our original department, as well as the substantial support rendered to our subscription list of late. To some of our new subscribers we have been unable to send the June number. The edition for July is also very nearly exhausted. That for August being larger than the preceding months, we hope to be able to furnish it to all new subscribers until October, by which time we expect to be warranted in a further increase of the edition.

---

*Geneva Medical College.*—We are requested to state that Prof. Wm. Sweetser has resigned the chair of Institutes and Practice of Medicine in this institution, and that Prof. Charles A. Lee is no longer connected with it.

---

Elisha Bartlett, M. D., the distinguished author and teacher, died in Rhode Island, on the 12th of July last.

